# Effects of Long-Term Fertilization and Stand Age on Root Nutrient Acquisition and Leaf Nutrient Resorption of *Metasequoia glyptostroboides*

**DOI:** 10.3389/fpls.2022.905358

**Published:** 2022-05-11

**Authors:** Rui Song, Ran Tong, Hui Zhang, G. Geoff Wang, Tonggui Wu, Xiuqing Yang

**Affiliations:** ^1^College of Forestry, Shanxi Agricultural University, Taigu, China; ^2^East China Coastal Forest Ecosystem Long-term Research Station, Research Institute of Subtropical Forestry, Chinese Academy of Forestry, Hangzhou, China; ^3^Forestry and Biotechnology College, Zhejiang A&F University, Hangzhou, China; ^4^Department of Forestry and Environmental Conservation, Clemson University, Clemson, SC, United States

**Keywords:** nutrient acquisition strategies, N and P fertilization, stand age, leaf, root

## Abstract

The plant nutrient acquisition strategies are diverse, such as root nutrient acquisition and leaf nutrient resorption, playing important roles in driving soil processes, vegetation performance as well as ecosystem nutrient cycling. However, it is still in a debate whether there is a synergy or tradeoff between above- and below-ground nutrient acquisition strategy under nitrogen (N) and phosphorus (P) addition, or with stand age. Herein, this study investigated the responses of root-soil accumulation factor (*RSAF*) and leaf nutrient resorption efficiency (*NuRE*) to long-term N and P fertilization, and further explored the trade-off between them in *Metasequoia glyptostroboides* plantations with different stand age. Results showed that under N fertilization in young plantations, leaf N resorption efficiency (*NRE*) increased, and root-soil accumulation factor for P (*RSAF-P*) decreased. For young forests under P fertilization, the *NRE* increased whereas *RSAF-P* decreased. For middle-aged forests under P fertilization, the *NRE* and leaf P resorption efficiency (*PRE*) increased and the *RSAF-P* decreased. Under P fertilization in young and middle-aged plantations, *PRE* had a significant positive correlation with *RSAF-P*. Under N fertilization in young plantations, *NRE* was significantly positive correlated with root-soil accumulation factor for N (*RSAF-N*). The covariance-based structural equation modeling (CB-SEM) analysis indicated that stand age had positive effects on *PRE* whether under N or P fertilization, as well as on *RSAF-P* under N fertilization, whereas had no effects on the *NRE* or *RSAF-N.* Overall, our results can shed light on the nutrient acquisition strategies of *M. glyptostroboides* plantations under future environmental changes and the results could be applied to the nutrient management practices.

## Introduction

The plant nutrient acquisition strategies cannot be underestimated in forest ecosystems, given that the plants usually do not have sufficient amounts of biologically available nutrients to support growth, survival, and reproduction, especially in nutrient-impoverished habitats (Abrahão et al., 2018; Wang et al., 2020). It has been always considered that roots are the major tissue for the plant to acquire nutrients, and root nutrient acquisition strategies play a decisive role in the maintenance of forest ecosystem health and vitality (Andersen et al., 2017; Ma et al., 2018; Li et al., 2019). In recent decades, some other plant nutrient acquisition strategies have also been proposed, such as leaf nutrient resorption proficiency and efficiency (Reed et al., 2012; He et al., 2020; Xu et al., 2020). Naturally, whether these plant nutrient acquisition strategies co-vary or exhibit coordinated responses to a changing environment has drawn great attention from ecologists.

Allocation of effort toward nutrient capture and resorption depends on both the environment nutrient availability and the cost involved in these processes (Wright and Westoby, 2003; Wang et al., 2014a). In barren soils, plants should bear traits that prioritize conservation over active absorption of resources, whereas the opposite was expected in nutrient-rich soils. Specifically, since nutrients derived from capture become less expensive than those from resorption as soil nutrient availability increases, the root capture strategy would be more favored than leaf resorption (Wright and Westoby, 2003; Kou et al., 2017). Inversely, species from nutrient-impoverished habitats also reduce nutrient losses by remobilizing a large fraction of the nutrients before senesced organ shedding (high remobilization efficiency), and shedding leaves with very low final nutrient concentrations (Killingbeck, 1996; Kobe et al., 2005; Hayes et al., 2014). Nevertheless, in cases of consistent environmental nutrient availability, the costs of the different plant nutrient acquisition strategies have not been effectively evaluated.

Increasing anthropogenic nitrogen (N) and phosphorus (P) deposition has enhanced N and P availability in many forest ecosystems, respectively, providing ideal venues for the studies of the trade-off relationships of plant nutrient acquisition strategies. It is well known that plants can maintain high nutrients level under nutrient enrichment through increasing above-ground nutrient conservation or improving below-ground nutrient uptake (Deng et al., 2016; Hofmann et al., 2016). Several studies showed the trade-off relationships between root nutrient acquisition and leaf nutrient resorption while only considering these two mechanisms (Deng et al., 2016; Kou et al., 2017). In contrast, other studies found the synergy relationship between these two mechanisms, and emphasized that only adopting above-ground nutrient conservation of below-ground nutrient uptake may not be applicative (Ushio et al., 2015; Hofmann et al., 2016).

The corresponding alterations in tree nutrient capture capacity during forest stand development had been widely demonstrated (Brant and Chen, 2015; Chen and Chen, 2022). For instance, the NRE rose and then dropped, and the PRE increased with stand age in *Medicago sativa*, which was closely related to the nutrient limitation of plant growth (Wang et al., 2014b). As trees had different root physiology and exudation patterns at different ages, their root nutrient capture capacity exhibited significant divergence (Liu et al., 2018). Yet, whether the patterns of the tree nutrient capture with different stand ages would alter under nutrient addition remained unclear.

As each mechanism demands distinct levels of resource investments, as well as its potential consequences for ecosystem functions, it is critical to understand how plants adjust above-ground conservation and below-ground uptake to alleviate N and P deficiency as stand development. Taken together, it is generally believed that a trade-off mechanism generally existed between the leaf nutrient resorption and the root acquisition strategies whether in the cases of sufficient or deficient nutrients supplies. In this study, we conducted N and P fertilization experiments to explore the effects of different gradient N and P fertilization on leaf nutrient resorption efficiency (*NuRE*) and root-soil accumulation factor (*RSAF*) in *M. glyptostroboides* plantations with different stand ages. Specifically, we aimed to: (1) describe the change trends of *NuRE* and *RSAF* with the increase of nutrient availability among nutrient fertilization types and along stand development; (2) explore the potential relationships between *NuRE* and *RSAF* under environmental changes.

## Materials and Methods

### Study Site

**FIGURE 1:**
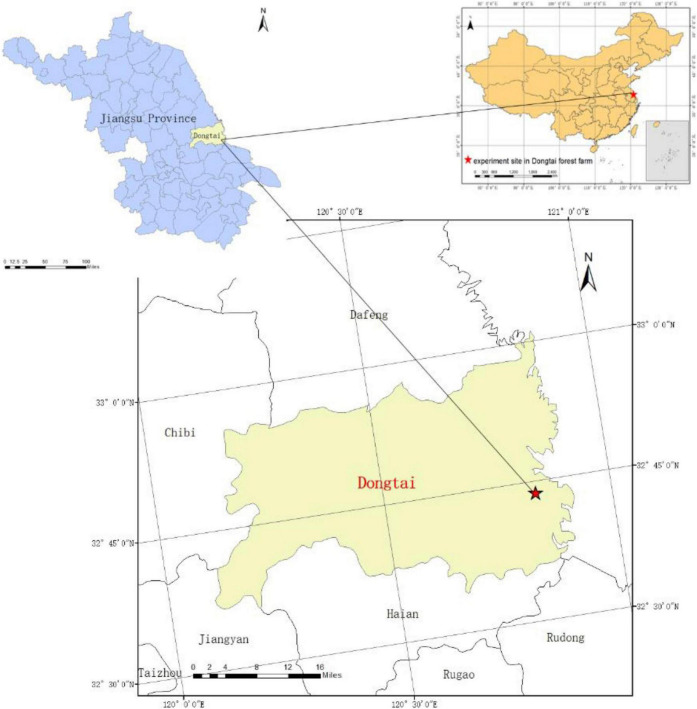
Location of *M. glyptostroboides* forest experiment site in Dongtai forest farm, Jiangsu Province, China.

The study was conducted in Dongtai Forest Farm (121° 45′E, 33° 42′N), a site located in the central coast of the Jiangsu Province, China. The climate of the study site is that of the monsoon subtropical moist marine climate zone. The altitude is 0–4 m, the annual average temperature, frost-free period, precipitation, and sunshine duration is 14.5°c, 220 days, 1055.7 mm, and 2130.5 h. The soil is alkaline sandy soil, which was more infertile than yellow-earth soil in the natural range of *M. glyptostroboides*. The study site was the pioneer area to plant the *M. glyptostroboides* in coastal China (Figure 1).

### Experiment Design and Sample Collection

N and P experiment was initiated in 2014 of different ages (young forest: 6-year old; middle-age forests: 24-year old) with three 300 m × 100 m plots of *M. glyptostroboides* plantations in Dongtai forest farm, and ten 20 m × 30 m plots of each plot were selected for completely random block N and P addition experiments, of which 5 plots were used for N addition treatments and 5 plots for P addition treatments in each age class. Each plot was separated by a buffer zone. The basic information is shown in Table 1. N and P addition gradients setting: set the N addition treatments as CK, 0.8, 2.4, 4.0, and 6.0 mol⋅m^–2^, respectively, and the fertilization was added in the form of urea [CO(NH_2_)_2_]; P fertilization treatment was set as CK, 0.05, 0.2, 0.6, and 1.0 mol⋅m^–2^, respectively, and fertilization was added in the form of superphosphate [Ca(H_2_PO_4_)_2_]. The fertilization gradients were based on a pretest and a previous seedling pot study (Wang et al., 2018). Fertilization dosage and time: the first addition time is from the end of March to the beginning of April, 60% of the total amount of fertilization added; the second addition time is in the middle of June, 40% of the total amount of fertilization added. In addition, N and P were applied to the field directly every year.

**TABLE 1:** General characteristics of the young and middle-aged *M. glyptostroboides* stands.

Basic properties	Parameter	Young plantations	Middle-aged plantations
Stand structure	Average height (m) DBH (cm) DBH (growth) Density (tree/hm^2^) Crown width (m)	10.33 ± 2.21 17.8 ± 0.86 4.8 ± 1.52 417 4.61 ± 0.65	15.91 ± 2.20 27.75 ± 0.84 2.39 ± 0.69 417 5.01 ± 0.52
Soil physical properties	Water content (%) Total porosity (%) Capillary porosity (%)	0.22 ± 0.02 44.09 ± 2.81 42.46 ± 3.68	0.32 ± 0.03 46.49 ± 2.39 45.75 ± 2.37
Soil chemical properties	Organic C content (g kg^–1^) Total N content (g kg^–1^) Available N content (mg kg^–1^) Total P content (g kg^–1^) Available P content (mg kg^–1^)	8.19 ± 0.38 0.71 ± 0.01 90.90 ± 4.27 0.85 ± 0.03 14.27 ± 3.91	13.18 ± 4.09 0.94 ± 0.08 132.67 ± 12.66 0.68 ± 0.10 2.85 ± 0.15

*The DBH (growth) is the DBH increasement during 2015–2019 year.*

Three average trees in each plot were selected for collecting leaves, branches, and roots. Fully expanded leaves and branches from the upper and outer part of tree crowns were sampled. The fine roots (<2 mm) of each selected individual were sampled through the careful removal of the soil surrounding the roots. Five soil cores (2.5 cm in diameter) per plot were randomly collected from 0 to 10 cm depth following the removal of understory plants and surface litter, and thoroughly mixed to homogenize a sample. Leaves, branches, roots, and soils were sampled in August 2018 (the end of the growing season). Five litter traps (1.0 m^2^, made of nylon mesh) per plot were fixed 1.0 m above the ground. Leaf litter was collected in late November 2018.

### Chemical Measurements

All plant organs samples were dried in the oven at 105°C for 2 h, then dried at 75°C to constant weight, crushed with a mechanical grinder, and the samples were sieved to determine nutrient content. The C and N concentrations were determined for each sample using an autoanalyzer (Kjeltec 2300 Analyzer Unit, Foss, Sweden). P concentration was determined using the standard ammonium molybdate method (reference code GBW08513; General Administration of Quality Supervision, PRC).

Soil samples were air-dried after being sieved (2-mm mesh). Soil pH was determined by the potentiometric method, soil organic carbon (SOC) was determined with wet oxidation by sulfuric acid and potassium dichromate and back titration with ferrous sulfate. The total N concentration was determined by Kjeldahl method, Hydrolysable N concentration was determined by titration with a dilute solution of H_2_SO_4_ after extraction with a mixture of ferrous sulfate and sodium hydroxide. The total P and available P concentrations were determined with a molybdate blue colorimeter after extraction with 0.5 M sodium bicarbonate (Zhang et al., 2018).

### Data Analysis

Nutrient resorption efficiency (NuRE) was defined as the proportional withdrawal of a nutrient during senescence and was calculated as follows:


$$N\,u\,R\,E=\left(1-\frac{N\,u_{s\,e\,n\,e\,s\,c\,e\,n\,c\,e\,d}}{N\,u_{g\,r\,e\,e\,n}}\times M\,L\,C\,F\right)\times 100\%$$

Where *NuRE* is N or P resorption efficiency, *Nu*_*senescenced*_ and *Nu*_*green*_ represent N or P concentration (mass-based) in leaf and litter, respectively, and *MLCF* is mass loss correction factor with a value of 0.745 for deciduous coniferous species (Vergutz et al., 2012).

Root-soil accumulation factor (*RSAF*) for N and P were defined to describe the root N or P acquisition (Kou et al., 2017), as follows:


$$R\,S\,A\,F=\frac{N\,u_{r\,o\,o\,t}}{N\,u_{s\,o\,i\,l}}$$

Where *RSAF* is root N or P accumulation factor, *Nu*_*root*_ and *Nu*_*soil*_ represent N or P concentration (mass-based) in absorptive fine root and soil, respectively.

Data analysis using the single factor variance (One-way ANOVA) analysis of the different fertilization gradients of plant organs stoichiometric characteristics, nutrient acquisition, nutrient recycling, and nutrient absorption efficiency under different stand ages, respectively, the influence of significance level set as *P* = 0.05, with minimal process significantly difference (LSD) determined the level of significance in SPSS 23.0 (SPSS Inc., Chicago, IL, United States). The covariance-based structural equation modeling (CB-SEM) analysis was conducted in SPSS Amos 26 (SPSS Inc., Chicago, IL, United States) to explore the effects of stand age on *NuRE* and *RSAF* under N and P fertilization. All data were tested to fulfill the assumptions of normality and homogeneity of variance, and transformations were carried out when necessary. Figures were plotted using Origin 2018 (Origin Lab Corporation, Northampton, MA, United States).

## Results

### Effect of Nitrogen and Phosphorus Fertilization on Leaf Nutrient Resorption Efficiency and Root-Soil Accumulation Factor

**FIGURE 2:**
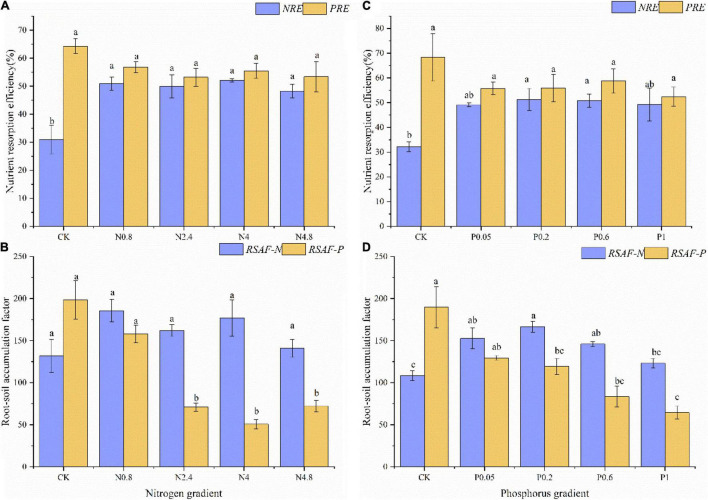
Effects of N fertilization (CK: control, N0.8: 0.8 mol N, N2.4: 2.4 mol N, N4: 4 mol N, N4.8: 4.8 mol N) on *NuRE* **(A)** and *RSAF* **(B)** in *M. glyptostroboides* forests of young forests. Values are presented as mean ± S.E. (*n* = 3). Effects of P fertilization (CK: control, P0.05: 0.05 mol P, P0.2: 0.2 mol P, P0.6: 0.6 mol P, P1: 1 mol P) on *NuRE* **(C)** and *RSAF* **(D)** in *M. glyptostroboides* forests of young forests. Values are presented as mean ± S.E. (*n* = 3). Different lowercase letters in the bars and above the bars indicated significant difference (*P* < 0.05), respectively.

Under N fertilization in young plantations, the *NRE* and *PRE* of CK treatment were 31.02 and 64.25%, respectively. The *NRE* remarkably increased while *PRE* kept relatively stable along N fertilization gradients (Figure 2A). The *RSAF-N* and *RSAF-P* of CK treatment were 131.75 and 198.49, respectively. The *RSAF-P* remarkably decreased while *RSAF-N* kept relatively stable along N fertilization gradients (Figure 2B).

Under P fertilization in young plantations, the *NRE* and *PRE* of CK treatment were 32.17 and 68.31%, respectively. The *NRE* remarkably increased, while PRE kept relatively stable along P fertilization gradients (Figure 2C). The *RSAF-N* and *RSAF-P* of CK treatment were 108.38 and 189.6, respectively. The *RSAF-N* and *RSAF-P* remarkably increased and decreased along P fertilization gradients (Figure 2D).

**FIGURE 3:**
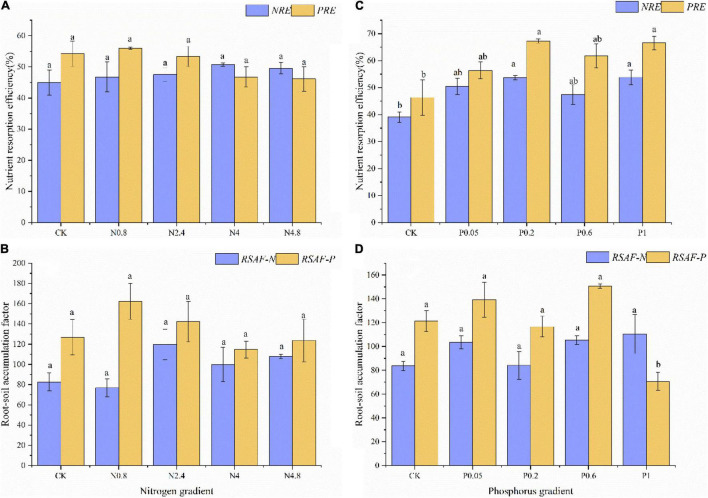
Effects of N fertilization (CK: control, N0.8: 0.8 mol N, N2.4: 2.4 mol N, N4: 4 mol N, N4.8: 4.8 mol N) on *NuRE* **(A)** and *RSAF* **(B)** in *M. glyptostroboides* forests of middle-aged forests. Values are presented as mean ± S.E. (*n* = 3). Effects of P fertilization (CK: control, P0.05: 0.05 mol P, P0.2: 0.2 mol P, P0.6: 0.6 mol P, P1: 1 mol P) on *NuRE* **(C)** and *RSAF* **(D)** in *M. glyptostroboides* forests of middle-aged forests. Values are presented as mean ± S.E. (*n* = 3). Different lowercase letters in the bars and above the bars indicated significant difference (*P* < 0.05), respectively.

Under N fertilization in middle-aged plantations, the *NRE* and *PRE* of CK treatment were 45.02 and 54.24%, respectively. The *NRE* and *PRE* both kept relatively stable along N fertilization gradients (Figure 3A). The *RSAF-N* and *RSAF-P* of CK treatment were 82.59 and 126.71, respectively. The *RSAF-N* and *RSAF-P* both kept relatively stable along N fertilization gradients (Figure 3B).

Under P fertilization in middle-aged plantations, the *NRE* and *PRE* of CK treatment were 39.07 and 46.33%, respectively. The *NRE* and *PRE* both remarkably increased remarkably along P fertilization gradients (Figure 3C). The *RSAF-N* and *RSAF-P* of CK treatment were 83.64 and 121.24, respectively. The *RSAF-N* and *RSAF-P* both kept relatively stable along P fertilization gradients (Figure 3D).

### Effect of Nitrogen and Phosphorus Fertilization on Leaf N Resorption Efficiency/leaf P Resorption Efficiency and Root-Soil Accumulation Factor for N/Root-Soil Accumulation Factor for P

**FIGURE 4:**
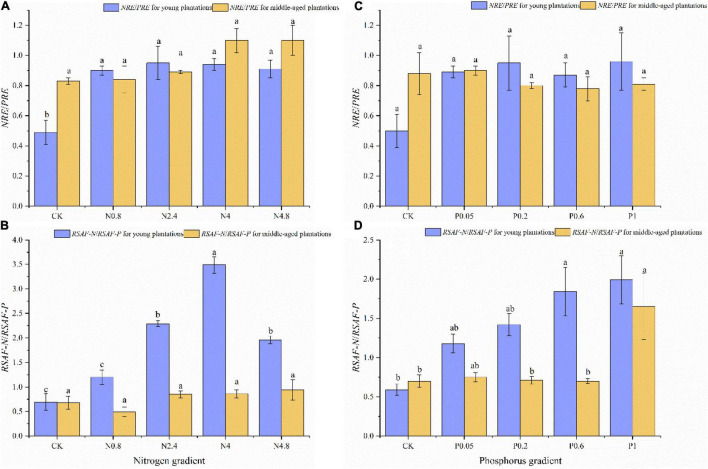
Effects of N fertilization (CK: control, N0.8: 0.8 mol N, N2.4: 2.4 mol N, N4: 4 mol N, N4.8: 4.8 mol N) on *NRE/PRE* **(A)** and *RSAF-N/RSAF-P* **(B)** in *M. glyptostroboides* plantations of different stand ages. Values are presented as mean ± S.E. (*n* = 3). Effects of P fertilization (CK: control, P0.05: 0.05 mol P, P0.2: 0.2 mol P, P0.6: 0.6 mol P, P1: 1 mol P) on *NRE/PRE* **(C)** and *RSAF-N/RSAF/P* **(D)** in *M. glyptostroboides* plantations of different stand ages. Different lowercase letters in the bars and above the bars indicated significant difference (*P* < 0.05), respectively.

The *NRE/PRE* of CK treatment was 0.49 and 0.83 in young and middle-aged plantations under N fertilization, respectively. The *NRE/PRE* for young plantation increased significantly under N fertilization, while the *NRE/PRE* for middle-aged plantations kept relatively stable along N fertilization gradients (Figure 4A). The *RSAF-N/RSAF-P* of CK treatment were 0.69 and 0.68 in young and middle-aged plantations under N fertilization, respectively. The *RSAF-N/RSAF-P* for young plantation increased significantly under N fertilization, while the *RSAF-N/RSAF-P* in middle-aged plantations kept relatively stable along N fertilization gradients (Figure 4B).

The *NRE/PRE* of CK treatment was 0.50 and 0.88 in young and middle-aged plantations under P fertilization, respectively. The *NRE/PRE* in young and middle-aged plantations both kept relatively stable along P fertilization gradients (Figure 4C). The *RSAF-N/RSAF-P* of CK treatment were 0.59 and 0.70 in young and middle-aged plantations under P fertilization, respectively. The *RSAF-N/RSAF-P* remarkably increased under P fertilization, while the *RSAF-N/RSAF-P* in middle-aged plantations kept relatively stable along P fertilization gradients (Figure 4D).

### The Relationships Between Leaf Nutrient Resorption Efficiency and Root-Soil Accumulation Factor Under Nitrogen and Phosphorus Fertilization

**FIGURE 5:**
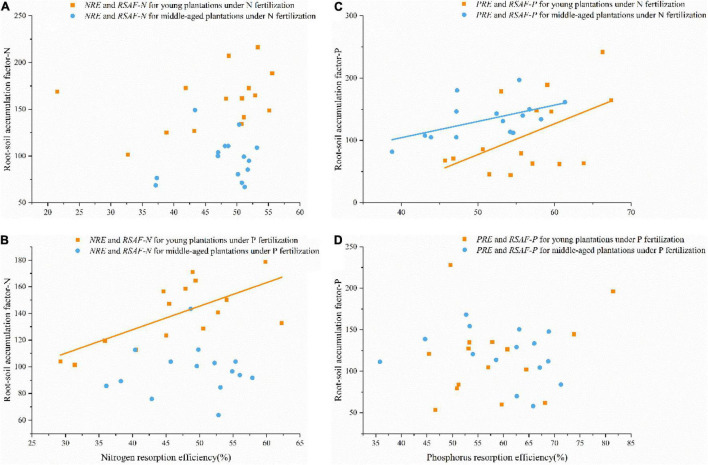
**(A)** Relationships between *NRE* and *RSAF-N* under N fertilization in young and middle-aged plantations (no significance). **(B)** Relationships between *NRE* and *RSAF-N* under P fertilization in young (*R*^2^ = 0.468, *P <* 0.01) and middle-aged (no significance) plantations. **(C)** Relationships between *PRE* and *RSAF-P* under N fertilization in young (*R*^2^ = 0.266, *P* < 0.05) and middle-aged plantations (*R*^2^ = 0.291, *P* < 0.05). **(D)** Relationships between *PRE* and *RSAF-P* under P fertilization in young and middle-aged plantations (no significance).

Under N fertilization, *NRE* showed no association with *RSAF-N*, while *PRE* was significantly positive correlated with *RSAF-P* in young and middle-aged plantations (Figures 5A,C). Under P fertilization, *NRE* was significantly positively correlated with *RSAF* for N in young plantation, whereas no significant relationships were found between *NRE* and *RSAF-N* in middle-aged plantation, as well as *PRE* and *RSAF-P* in both young and middle-aged plantations (Figures 5B,D).

### The Direct and Indirect Effect of Nutrient Fertilization and Stand Age on Leaf Nutrient Resorption Efficiency and Root-Soil Accumulation Factor

**FIGURE 6:**
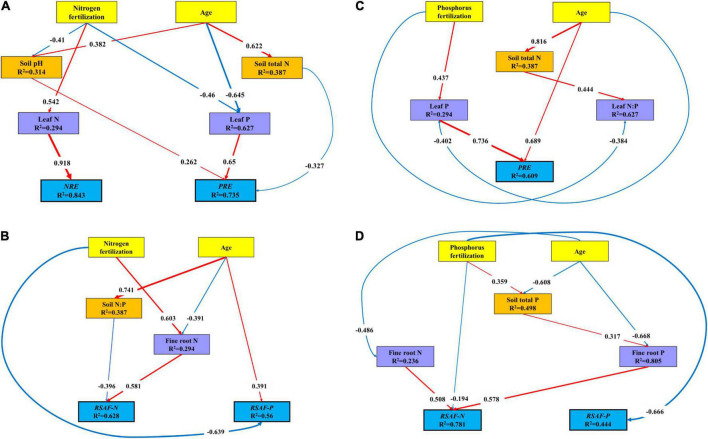
Covariance-based structural equation modeling (CB-SEM) analysis of the direct and indirect effects among variables. All the paths in the model were significant, and the standardized coefficients were listed on them. The thickness of the solid arrows reflected the magnitude of the standardized SEM coefficients, the red solid line represented the positive effect while the blue solid line represented the negative effect. **(A)** Results of model fitting: *X*^2^ = 13.51; Probability level = 0.563; df = 15; GFI = 0.9; RMSEA = 0.000; AIC = 55.51. **(B)** Results of model fitting: *X*^2^ = 7.116; Probability level = 0.524; df = 8; GFI = 0.927; RMSEA = 0.000; AIC = 33.116. **(C)** Results of model fitting: *X*^2^ = 5.915; Probability level = 0.55; df = 7; GFI = 0.938; RMSEA = 0.000; AIC = 33.915. **(D)** Results of model fitting: *X*^2^ = 10.519; Probability level = 0.484; df = 11; GFI = 0.918; RMSEA = 0.000; AIC = 44.519.

The covariance-based structural equation modeling (CB-SEM) analysis indicted that N fertilization and stand age together explained 84.3% of variations in *NRE* and 73.5% of variations in *PRE* (Figure 6A). N fertilization had a positive indirect effect on *NRE via* leaf N, and a negative indirect effect on *PRE* through leaf P and soil pH. Stand age had a positive direct effect on *PRE via* soil pH, and a negative indirect effect *via* soil total N and leaf P. N fertilization and stand age together explained 62.8% of variations in *RSAF-N* and 56.0% of variations in *RSAF-P* (Figure 6B). N fertilization and stand age had a negative and positive direct effect on *RSAF-P*, respectively. N fertilization had a positive indirect effect on *RSAF-N via* fine root N. Stand age had a negative indirect effect on *RSAF-N via* soil N:P.

P fertilization and stand age together explained 60.9% of variations in PRE (Figure 6C). Stand age had a positive direct effect on PRE, and P fertilization had a positive indirect effect on PRE *via* leaf P. P fertilization and stand age together explained 78.1% of variations in *RSAF-N*, and 44.4% of variations in *RSAF-P* (Figure 6D). P fertilization had a negative direct effect on *RSAF-P*, and stand age had a negative direct effect on *RSAF-N*. Stand age had a negative indirect effect on *RSAF-N via* fine root N and P.

## Discussion

### Effects of Nitrogen Fertilization on Plant Nutrient Acquisition

Bioavailable N is increasing due to human activity, which greatly alters nutrient availability for plant growth and affects fundamental ecological processes (Wang et al., 2019a). Nutrient resorption from senescing leaves is one of the plants’ essential nutrient conservation strategies, accounting for a large property of the nutrient demand for plants (Aerts, 1996). In this study, N fertilization increased leaf N concentration in young plantation, which was in line with the observations from the global-scale meta-analysis (Ostertag and DiManno, 2016; Sardans et al., 2017). However, in contrast to most previous studies, we found a significant increase in *NRE* for young plantation and no significant changes for middle-aged plantation under N fertilization (Yuan and Chen, 2015; Lu et al., 2019; Zhao et al., 2020). Our study also found that leaf litter N concentration kept relatively stable under N fertilization whether in young and middle-aged plantations (Wen et al., 2021). Thus, considering the violent strong natural and human-activities disturbances, we deemed that nutrient return of leaf litter to the soil, i.e., litter decomposition, might not be so essential, especially in the coastal man-made forests. Moreover, the *RSAF-P* decreased remarkably under N fertilization, which was not consistent with Kou et al. (2017), who reported that the *RSAF-P* kept relatively stable in *Pinus elliottii* plantations.

The *NRE*/*PRE* in leaf was recently developed to indicate the theoretical framework of N or P limitation for plant growth, which could be applied to quantitatively evaluate the spatial characteristics and key influencing factors of N and P limitation in the terrestrial ecosystems (Du et al., 2020). In this study, the *NRE/PRE* was always less than 1 under N fertilization, indicating that the plant growth was mostly limited by N. This result was consistent with one of our previous studies, which found N was the main limiting factor for *M. glyptostroboides* growth combining with the concept of stoichiometric homeostasis (Wang et al., 2019b). Moreover, the *NRE/PRE* of young plantation increased along the gradient of N fertilization, demonstrating that the N limitation got relieved to some extent (Li et al., 2016; Du et al., 2020).

It was always expected that plant tissues exhibited trade-offs relationships in water and nutrient acquisition, which is of great significance for plant stoichiometric homeostasis and the balance of matters and energy in the terrestrial ecosystems. While these trade-off relationships became unpredictable under the background of global change. In this study, we observed significant and positive associations between *PRE* and *RSAF-P* in both young and middle-aged plantations under N fertilization, which was not in line with Kou et al. (2017) for *Pinus elliottii* plantations. However, most of the trade-off relationships between leaf nutrient resorption and fine root nutrient acquisition had been lost under N fertilization. Therefore, we referred that the changes in environmental nutrient conditions might lead to the absence of trade-off relationships between under- and above-ground plant tissues.

### Effects of Phosphorus Fertilization on Plant Nutrient Acquisition

Atmospheric P deposition brought by agricultural activities, dust transport, and combustion source emissions had greatly altered the soil P availability that greatly influenced plant growth (Widdig et al., 2019; Pan et al., 2021). Generally, the availability of one nutrient would inevitably affect relative availability of other nutrients, particularly in the status of nutrient deficiency or imbalance (Kou et al., 2017). In the current study, the *NRE* of young and middle-aged plantation increased along the gradient of P fertilization, which was also reported in Yan et al. (2015). Furthermore, the *RSAF-N*/*RSAF-P* increased remarkably whether under P fertilization in young plantation, and kept relatively stable in middle-aged plantation. This finding was also in line with the above observations for above-ground plant tissues. Therefore, fine root nutrient acquisition might play an important role in indicating the nutrient limitation of plant growth. Meanwhile, we observed that the *RSAF-P* decreased significantly under P fertilization whether in young or middle-aged plantation, which might be attributed to the fact that the fine root nutrient acquisition existed some delayed effects. Meanwhile, some limitations appeared in this study such as considering the fine roots with a diameter less than 2 mm, ignoring the high morphological and functional heterogeneity in fine roots (Pregitzer et al., 2002; Kou et al., 2018), which might lead to an underestimate of the *RSAF.*

Notably, the *NRE* and *RSAF-N* in young plantation under P fertilization, were significantly correlated with each other, even exhibiting synergy relationships to some extent. These findings were also observed in previous research, which emphasized that only adopting aboveground N and P conservation or belowground N and P uptake might not be accurate (Ushio et al., 2015; Hofmann et al., 2016). Therefore, we concluded that the synergistic relationship for P acquisition might be explained by the two following reasons. Firstly, the relationships between fine root nutrient acquisition and leaf nutrient resorption were indeed positively related, whereas there might exist some other mechanisms which could balance these two nutrient conversation mechanisms. Secondly, there was no trade-off evolutionary mechanism between leaves and roots, which might leading to the consistent responses of leaf nutrient resorption and fine root nutrient acquisition to environment changes by chance.

### Stand Age Mediates Fertilization Effects on Plant Nutrient Acquisition

Trees at different growth stages have great differences in physiological processes and nutrition requirements, resulting in changes of nutrient acquisition strategies along an age sequence (Sun et al., 2016; Zhang et al., 2018). However, most previous studies considered the stand age effects on nutrient acquisition strategies in natural conditions, while little was known about its combination with environmental changes. In the current study, we used SEM analysis to explore the direct and indirect effects of stand age on *NuRE* and *RSAF* under N and P fertilization. Generally, stand age had positive effects on *PRE* whether under N or P fertilization, as well as on *RSAF-P* under N fertilization, which indicated that plant intended to resorb more P from senescing leaves and soil by fine root to deal with strengthening P-limitation during stand development (Zhang et al., 2018; Wang et al., 2019b). Thus, these findings confirmed that stand age mediated the fertilization effects on nutrient acquisition strategies.

## Conclusion

This study evaluates how N and P fertilization affects nutrient acquisition strategies in *M. glyptostroboides* plantations with different stand ages of the coastal in China. We found that N fertilization increased *NRE*, while decreased *RSAF-P* in young plantations. P fertilization increased *NRE* and *PRE*, while decreased *RSAF-P* in middle-aged plantations. Little synergetic relationships were observed between *NuRE* and *RSAF* whether under N and P fertilization, as well as in young and middle-aged plantations. Stand age had positive effects on *PRE* and *RSAF-P*, whereas had no effects on the *NRE* or *RSAF-N.* These findings provided new insights into the predictions for the facts that how variations in nutrient availability induced by the global change will influence plant nutrient uptake and nutrient conservation strategies.

Please confirm that the Data Availability statement is accurate. Note that we have used the statement provided at Submission. If this is not the latest version, please let us know. Note that we have used the statement provided at Submission. If this is not the latest version, please let us know.

## Data Availability Statement

The original contributions presented in the study are included in the article/supplementary material, further inquiries can be directed to the corresponding author/s.

## Author Contributions

TW and XY designed the project and participated in the manuscript. RS, RT, and GW wrote and revised the manuscript. RS and HZ analyzed the data and constructed the database. RS collected the samples. All authors revised and approved the manuscript.

## Conflict of Interest

The authors declare that the research was conducted in the absence of any commercial or financial relationships that could be construed as a potential conflict of interest.

## Publisher’s Note

All claims expressed in this article are solely those of the authors and do not necessarily represent those of their affiliated organizations, or those of the publisher, the editors and the reviewers. Any product that may be evaluated in this article, or claim that may be made by its manufacturer, is not guaranteed or endorsed by the publisher.
